# EGFR polymorphisms drive lung cancer risk and survival disparities: a genotype-expression-outcome cohort study

**DOI:** 10.3389/fgene.2025.1591539

**Published:** 2025-05-14

**Authors:** Chao Zuo, Ziqiang Wang, Yi Liu, Jing Cheng, Dongli Yang, Yu Wang, Yongchao Qiao

**Affiliations:** ^1^ Department of Clinical Laboratory, The First Affiliated Hospital of Guilin Medical University, Guilin, Guangxi, China; ^2^ Research Center of Clinical Laboratory Science, Bengbu Medical University, Bengbu, Anhui, China; ^3^ Department of Geriatrics, The First Affiliated Hospital of Guilin Medical University, Guilin, Guangxi, China

**Keywords:** EGFR, polymorphism, lung cancer, susceptibility, prognosis

## Abstract

**Purpose:**

To investigate the correlation between single-nucleotide polymorphisms (SNPs) of the Epidermal growth factor receptor (EGFR) gene and its protein expression with susceptibility and survival prognosis of lung cancer (LC) patients.

**Methods:**

Using SNP-scan high-throughput technology, the EGFR gene’s rs2227983, rs2293347, and rs884225 locations were analyzed in 300 LC patients and 150 healthy individuals. And small cell lung cancer (SCLC), lung adenocarcinoma (LUAD), and lung squamous carcinoma (LUSC) were subdivided into groups for lung cancer patients. Chi-square test and logistic regression analysis were used to assess the susceptibility of LC. The correlation between SNP haplotypes and LC risk was analyzed using the SHEsis website. KM curves and Cox regression were used to analyse the association between polymorphisms and survival prognosis of LC patients. Expression differences in protein levels were analyzed using immunohistochemistry.

**Results:**

EGFR rs2293347 was associated with LUAD, LUSC, and SCLC susceptibility, and rs884225 was associated with LUAD susceptibility. Haplotype ATT was associated with LC and histological type LUAD and SCLC susceptibility. Meanwhile, rs2293347-TT and rs884225-TT were associated with worse prognosis, and rs2293347-TT was an independent risk factor for prognosis in patients with LC. Furthermore, tumor tissue EGFR protein levels were elevated in patients with both genotypes.

**Conclusion:**

EGFR rs2293347 (pan-subtype) and rs884225 (LUAD-specific) polymorphisms increase LC risk through elevated protein expression, with rs2293347-TT conferring worse survival. These genotype-protein correlations highlight their dual role as susceptibility markers and prognostic predictors in precision oncology.

## 1 Introduction

The latest annual global cancer report, released in 2022, ranked lung cancer (LC) as the second most prevalent cancer in the world and the first in terms of mortality (Siegel et al., 2022). At present, the pathogenesis of LC has not been clearly defined. Existing studies have shown that it is not only related to smoking, radiation and other factors, but also related to family genetics, immune function, endocrine metabolism, gene mutation and other factors, which is the product of the interaction between environmental factors and genetic factors (Huang et al., 2021; Malhotra et al., 2016; Krabbe et al., 2024).

Epidermal growth factor receptor (EGFR; also known as HER-1 or ErbB-1) is a transmembrane glycoprotein receptor for cell proliferation and signal transduction (Voldborg et al., 1997). Upon binding to ligands such as epidermal growth factor (EGF) (Wang et al., 2018) and transforming growth factor alpha (TGF-α) (Lin et al., 2020), it induces the formation of a dimer between two adjacent EGFR monomers, leading to inhibite EGFR tyrosine kinase structural domain activation and autophosphorylating EGFR residues, triggering a variety of downstream signaling pathways essential for the regulation of cell proliferation, differentiation, migration, invasion, metastasis and survival, including protein kinase B (PKB), stress-activated protein kinase (SAPK), and mitogen-activated Protein Kinase (MAPK). Activated protein kinase could affect normal cellular physiological processes or induce the generation and exacerbation of malignant biological behaviours (Wu and Shih, 2018; Lim et al., 2018; Oxnard et al., 2011).

Currently, more studies have highlighted that high expression of EGFR is associated with worse prognosis in various cancers, such as cervical cancer (Kim et al., 2002), bladder cancer (Colquhoun and Mellon, 2002). It remains controversial whether the EGFR pathway plays a role in the prognosis of non-small cell lung cancer (NSCLC) (Liu et al., 2017), while combined Retinoblastoma 1 (RB1) and Tumor Protein 53 (P53) mutations lead to poorer clinical outcomes in small cell lung cancer (SCLC) (Solta et al., 2024). EGFR polymorphisms, as a form of genetic variation in addition to EGFR mutations, have been reported to correlate with the prognosis of survival in patients with EGFR mutation-positive LC treated with tyrosine kinase inhibitors (TKIs) (Oxnard et al., 2011; Leonetti et al., 2019; He et al., 2021), and some studies (Bashir et al., 2019; Liu et al., 2015; Feng et al., 2014; Li et al., 2023; Bashir et al., 2018) have shown an association with susceptibility in LC patients. Of these, rs2227983 is thought to be associated with TKIs toxicity (Obradovic et al., 2023) and is associated with NSCLC risk in multiple populations (Bashir et al., 2019; Winther-Larsen et al., 2019; Zhang et al., 2013; Winther-Larsen et al., 2015; Ma et al., 2009), but it has also been shown to be unrelated to the susceptibility and prognosis of NSCLC treated with TKIs (Jurisic et al., 2020). Therefore, the results of these studies have not been harmonised and do not take into account the histological types and clinical characteristics of LC patients and the association with prognosis. This study aimed to investigate the potential correlation of EGFR gene polymorphisms and their protein levels interaction with the susceptibility, prognosis, and various clinical indicators of LC patients.

## 2 Materials and methods

### 2.1 Subjects and ethics of study

This research included 300 individuals identified with LC at The First Affiliated Hospital of Guilin Medical University’s Oncology Department between July 2021 and March 2023, categorized into LC groups based on histopathological types, with 160 in LUAD, 77 in LUSC, and 63 in SCLC. The diagnostic and pathological classification criteria of LC were based on the Chinese Society of Clinical Oncology (CSCO) 2022 Guidelines for the Diagnosis and Treatment of Primary Lung Cancer, and pathological examination (including cytology, histology, immunohistochemistry, lymph node, bronchoscopy, and lung puncture biopsy, etc.) was taken as the gold standard for the diagnosis of LC. Pathological classification of LC was based on the WHO Histological Classification of Lung Tumor (5th edition, 2021) issued by the World Health Organization (WHO). At the same time, 150 healthy volunteers were randomly recruited as healthy controls (HC group) from the health check-up centre based on age and gender of inclusion. Individuals suffering from acute liver or kidney issues, past or present autoimmune conditions, and those who were pregnant were not included. The participants were independent persons in Guangxi and had no familial ties to one another. Guilin Medical University’s Affiliated Hospital’s Medical Ethics Committee examined and sanctioned the study’s protocol (No. 2022YJSLL-78), ensuring compliance with the principles of the Declaration of Helsinki. Every participant offered to take part in this research and provided their signed informed consent. Overall Survival (OS) The outcome indicator is time to death; this death is any death from any cause and is counted. Progression Free Interval (PFI) is the period of survival without further deterioration of the disease after treatment. The outcome indicator is the occurrence of deterioration (re-admission to hospital).

### 2.2 DNA extraction and genotyping of the EGFR polymorphism

The workflow commenced with locus-specific probe design for SNP analysis. Subsequent steps included DNA sample preparation (30–50 ng/μL) via thermal lysis (98°C for 5 min) and probe hybridization under denaturation-renaturation conditions. Sequential reactions were then executed: (1) ligation (94°C/1°min→58°C/4 h) with optimized reagent ratios (0.5 μL ligase: 1 μL probe), (2) multiplex fluorescent PCR amplification (touchdown cycles: 62→57°C, 25 standard cycles) using precise primer-mix stoichiometry (10:1 PCR mix: primer), and (3) ABI3730XL sequencing of diluted amplicons (×10) with Liz600 size standard. Allelic discrimination was ultimately achieved through GeneMapper 4.1 analysis of raw electrophoretic data. Supplementary Table S1 provides the PCR primer sequences for the EGFR gene SNPs rs2227983, rs2293347, and rs884225. These SNPs with suballele mutation rates >5% in Asian populations from the NCBI SNP database.

### 2.3 Baseline information and testing indicators

The liver and kidney function indexes of each study subject were completed in this laboratory, and every testing method adhered rigorously to the guidelines of the reagent kit and the manual for instrument operation, utilizing either the Roche Cobas E701 or E801 analyzer, which was certified by ISO15189 (NO.ML00036). Blood samples analyzed in this study were collected concurrently with SNP sequencing to ensure batch consistency.

### 2.4 IHC (immunohistochemistry)

Human tissue experiments received approval from the First Affiliated Hospital of Guilin Medical University’s Institutional Review Board (No. 2022YJSLL-78), adhering to the Helsinki Declaration’s guidelines. Utilized protocols for primary antibody and antigen recovery included: anti-EGFR (CER-0032, MXB, Fuzhou, China). Three patients with each histological type of lung cancer were randomized to each genotype at each of the three SNPs, for a total of 81 patients. Two full senior pathologists assessed positive indicators and degree interpretation. The staining results were classified into four grades from 0 to 3+, with the following criteria: 0 as no staining; 1+ as light yellow staining of tumour cells without obvious granules or no more than 10% of tumour cells with yellow staining with obvious granules; 2+ as more than 10% of tumour cells with yellow staining with obvious granules or no more than 10% of tumour cells with brown staining with obvious granules; and 3+ as more than 10% of tumour cells with brown staining with obvious granules. The IOD/area ratio was calculated using ImageJ.

### 2.5 Statistical analysis

Statistical analyses for this study were completed using IBM SPSS 27.0 and R (4.2.1). Count data were described by frequency (n) or frequency [n (%)], and comparisons of count data and identification of Hardy-Weinberg equilibrium (HWE) law were carried out using Pearson’s chi-square test and chi-square test for goodness of fit in that order. Measurement data were validated for normality using the Kolmogorov-Sminov test; conformity to normal distribution was described as mean ± standard deviation, comparisons between two groups were performed using the independent samples *t*-test, and one-way ANOVA analysis of variance was used between multiple groups; non-conformity to normal distribution was described as median (quartile) [M (P25∼P75)], comparisons between two groups were performed using the Mann-Whitney U-test, and between multiple groups Kruskal–Wallis test was used. The relationship between biochemical markers, genetic SNP models, and LC susceptibility was evaluated using binary logistic regression, leading to the computation of odds ratio (OR) and 95% confidence interval (CI). One-way multifactor cox regression analyses and survival curves (also known as Kaplan-Meier curves) were performed using R software and the survival package [3.3.1], with the survminer package [0.4.9] and ggplot2 [3.3.6] used for visualization (variables with *P*-value < 0.1 in univariate analyses were enrolled in multivariate Cox models). The Tukey *post hoc* test was used for the analysis of protein expression differences between genotypes. *P* < 0.05 was considered statistically significant.

## 3 Results

### 3.1 Comparison of clinical and biochemical characteristics

Table 1 summarizes the clinical and biochemical characteristics of HC and LC. Results indicated a notable deviation in liver and kidney function indices in LC patients relative to HC (*P* < 0.001), highlighting a substantial impairment in these functions in LC patients.

**TABLE 1:** Comparison of clinical information of the study subjects.

Variables	HC (*n* = 150)	LC (*n* = 300)	*χ* ^2^/*Z*/*t*	*P*-value
N (M:F)	102/48	197/103	0.244	0.621^a^
Age (years)	59 (52.75–66)	61 (56–67)	−1.867	0.062^c^
TBIL (μmol/L)	11.1 (8.6–14.1)	6.3 (4.4–8.58)	−11.421	**<0.001** ^**c**^
DBIL (μmol/L)	4.1 (3.3–4.83)	2.8 (2.13–3.7)	−8.799	**<0.001** ^**c**^
IBIL (μmol/L)	7 (5.3–9.2)	3.17 (2.2–4.98)	−12.116	**<0.001** ^**c**^
TP (g/L)	73.69 ± 3.75	72.41 ± 6.15	2.737	**0.006** ^**b**^
ALB (g/L)	45.15 (43.7–46.6)	39.2 (36.7–42.38)	−13.599	**<0.001** ^**c**^
GLO (g/L)	29 (26.88–31.03)	32.15 (29.28–36.35)	−8.684	**<0.001** ^**c**^
A/G	1.57 (1.44–1.7)	1.22 (1.03–1.39)	−12.897	**<0.001** ^**c**^
ALP (U/L)	68 (58.75–81)	82 (66–107)	−6.253	**<0.001** ^**c**^
ALT (U/L)	18.4 (13.78–25.73)	16.15 (11.03–24.9)	−2.648	**0.008** ^**c**^
AST (U/L)	19.1 (16.05–23.33)	21.1 (16.63–27.58)	−2.899	**0.004** ^**c**^
LDH (U/L)	159.5 (146–184.25)	221.5 (182–260.75)	−11.033	**<0.001** ^**c**^
BUN (mmol/L)	5.1 (4.3–6.3)	5.15 (3.93–6.38)	−0.463	0.644^c^
Cr (μmol/L)	80.5 (68.75–90)	72 (60–85.75)	−4.028	**<0.001** ^**c**^
UA (μmol/L)	359 (307.5–440)	325 (257.25–394.5)	−3.907	**<0.001** ^**c**^
TCO_2_ (mmol/L)	25.26 ± 1.75	24.57 ± 2.74	3.231	**0.001** ^**b**^
GFR (mL/min)	82.95 ± 19.66	70.44 ± 18.48	6.622	**<0.001** ^**b**^
Cys-C (mg/L)	0.95 (0.84–1.1)	1.09 (0.94–1.25)	−6.025	**<0.001** ^**c**^

Data with normal distribution was indicated by mean ± standard deviation (SD), otherwise, it was presented by median (inter-quartile range, P25-P75). The *P*-values were calculated by ^a^Pearson chi-square test, ^b^Independent-sample *T*-test, and ^c^Mann-Whitney U test, separately. Bold value indicates statistical significance.

### 3.2 Relationship between EGFR gene polymorphism and LC susceptibility

The results of multiple fluorescence PCR typing of the EGFR gene of rs2227983, rs2293347, and rs884225 are shown in Supplementary Figure S1, in which the pure genotypes are single peaks and the heterozygous genotypes are double peaks. Hardy-Weinberg’s law of genetic equilibrium of HC, LC, and population size (*P* > 0.05) was in agreement with these three SNPs, indicating that selected subjects were well represented in the population. The rs2293347 CC genotype and C allele frequencies were higher in the LC patients than in the HC population, and the CT and TT genotypes and T allele frequencies were lower than in the HC population (*χ* ^2^ = 12.794 and 9.803, both *P* = 0.002, Table 2). Notable variances were observed in the rs2293347 genotype and allele frequency among the LUAD, LUSC, and SCLC groups versus the HC group (all *P* < 0.05, Table 3), along with a marked disparity in allele frequency at the rs884225 in the LUAD group versus the HC group (*P* = 0.024, Table 3).

**TABLE 2:** EGFR gene polymorphism in Chinese patients with lung cancer.

SNP	Genotype and allele	HC	LC	*χ* ^2^	*P*-value
rs2227983	GG	34 (22.7)	71 (23.7)	2.565	0.277
	GA	68 (45.3)	154 (51.3)		
	AA	48 (32.0)	75 (25.0)		
	G	136 (45.3)	296 (49.3)	1.282	0.258
	A	164 (54.7)	304 (50.7)		
rs2293347	CC	59 (39.3)	171 (57.0)	12.749	**0.002**
	CT	71 (47.3)	97 (32.3)		
	TT	20 (13.3)	32 (10.7)		
	C	189 (63.0)	439 (73.2)	9.803	**0.002**
	T	111 (37.0)	161 (26.8)		
rs884225	TT	49 (32.7)	85 (28.3)	2.909	0.234
	TC	80 (53.3)	154 (51.3)		
	CC	21 (14.0)	61 (20.3)		
	T	178 (59.3)	324 (54.0)	2.306	0.129
	C	122 (40.7)	276 (46.0)		

EGFR, Epidermal growth factor receptor; HC, Health control; LC, Lung cancer. Date are shown as n(percent). The P values were calculated by chi-square test. Bold value indicates statistical significance.

**TABLE 3:** Comparison of EGFR genotype and allele frequency distribution among subgroups.

Genotype and allele	HC	LUAD	LUSC	SCLC	*χ* ^2^	*P* ^*a*^	*χ* ^2^	*P* ^*b*^	*χ* ^*2*^	*P* ^*c*^
rs2227983										
GG	34 (22.7)	39 (24.4)	19 (24.7)	13 (20.6)	4.744	0.093	0.162	0.922	0.533	0.766
GA	68 (45.3)	87 (54.4)	35 (45.5)	32 (50.8)						
AA	48 (32.0)	34 (21.3)	23 (29.9)	18 (28.6)						
G	136 (45.3)	165 (51.6)	73 (47.4)	58 (46.0)	2.405	0.121	0.175	0.675	0.017	0.895
A	164 (54.7)	155 (48.4)	81 (52.6)	68 (54.0)						
rs2293347										
CC	59 (39.3)	94 (58.8)	43 (55.8)	34 (54.0)	12.341	**0.002**	6.268	**0.044**	6.433	**0.040**
CT	71 (47.3)	55 (34.4)	24 (31.2)	18 (28.6)						
TT	20 (13.3)	11 (6.9)	10 (13.0)	11 (17.5)						
C	189 (63.0)	243 (75.9)	110 (71.4)	86 (68.3)	12.267	**<0.001**	3.215	0.073	1.070	0.301
T	111 (37.0)	77 (24.1)	44 (28.6)	40 (31.7)						
rs884225										
TT	49 (32.7)	40 (25.0)	25 (32.5)	20 (31.7)	6.000	**0.050**	0.733	0.693	0.107	0.948
TC	80 (53.3)	81 (50.6)	38 (49.4)	35 (55.6)						
CC	21 (14.0)	39 (24.4)	14 (18.2)	8 (12.7)						
T	178 (59.3)	161 (50.3)	88 (57.1)	75 (59.5)	5.085	**0.024**	0.201	0.654	0.001	0.971
C	122 (40.7)	159 (49.7)	66 (42.9)	51 (40.5)						

EGFR, Epidermal growth factor receptor; HC, Health control; LUAD, lung adenocarcinoma; LUSC, lung squamous carcinoma; SCLC,small cell lung cancer. Date are shown as n(percent). The P values were calculated by chi-square test. P^a^: HC vs. LUAD; P^b^: HC vs. LUSC; P^c^: HC vs. SCLC. Bold value indicates statistical significance.

Genetic regression models showed that rs2293347CT and TT + CT genotypes were 0.471 and 0.489 times more susceptible to the risk of LC than the CC genotype, respectively (OR = 0.471 and 0.489, 95% CI = 0.308–0.722 and 0.328–0.729, both *P* < 0.001), the T allele was 0.624 times more likely to be susceptible to LC than the C allele (OR = 0.624, 95% CI = 0.465–0.839, *P* = 0.002), and the CC + TT genotype was 1.881 times more likely to be susceptible to LC than the CT genotype (OR = 1.881, 95% CI = 1.259–2.810, *P* = 0.002).

LUAD subgroup analysis showed that the rs2227983 GG + GA genotype was 1.744 times more susceptible to LUAD risk than the AA type (OR = 1.744, 95% CI = 1.046–2.907, *P* = 0.033); and that the rs2293347 CT, TT, and TT + CT genotypes were 0.486, 0.345, and 0.455 times (OR = 0.486, 0.345, 0.455, 95% CI = 0.301–0.785, 0.154–0.772, 0.289–0.717, *P* = 0.003, 0.010, *P* < 0.001), and the T allele was 0.540 times more common than the C allele (OR = 0.540, 95% CI = 0.381–0.764, *P* < 0.001), the CC + TT genotype was 1.716 times more common than the CT type (OR = 1.716, 95% CI = 1.086–2.711, *P* = 0.021); rs884225 CC genotype was 2.275 times more common than the TT type (OR = 2.275, 95% CI = 1.158–4.469, *P* = 0.017), the C allele was 1.441 times more common than the T allele (OR = 1.441, 95% CI = 1.048–1.980, *P* = 0.024), and the TT + TC genotype was 0.505 times more common than the CC phenotype (OR = 0.505, 95% CI = 0.281–0.907, *P* = 0.022).

LUSC subgroup analysis showed that rs2293347 CT and TT + CT genotypes were 0.464 and 0.513 times more likely to be susceptible to LUSC than the CC genotype (OR = 0.464 and 0.513, 95% CI = 0.253–0.851 and 0.294–0.894, *P* = 0.013 and 0.019 respectively), and the CC + TT genotype was 1.985 times more likely to be susceptible than the CT genotype (OR = 1.985, 95% CI = 1.112–3.541, *P* = 0.020).

**FIGURE 1:**
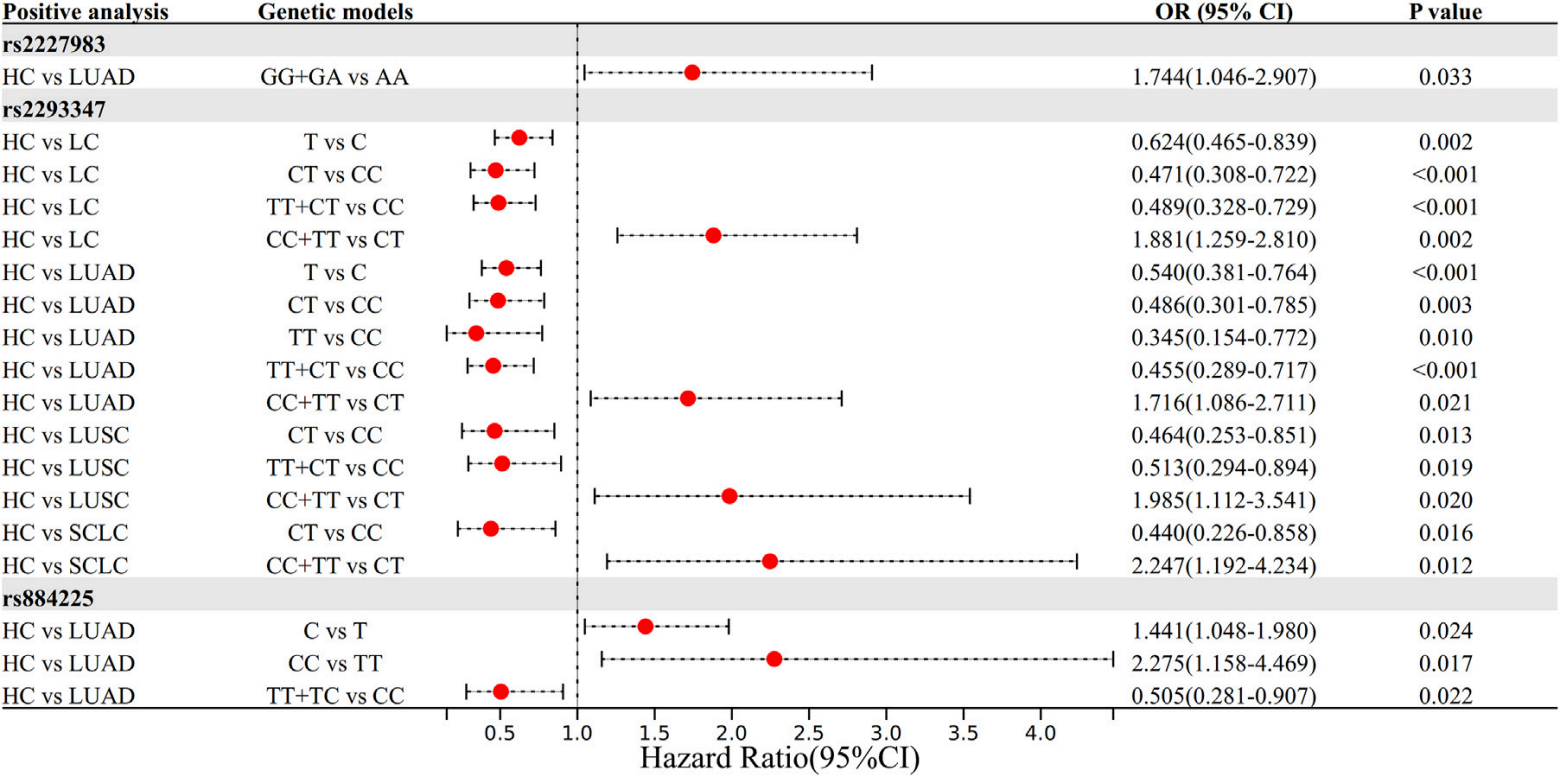
The forest plot of positive results in the Logistic regression analysis.

SCLC subgroup analysis showed that the rs2293347 CT genotype was 0.440 times more likely to be susceptible to SCLC than the CC genotype (OR = 0.440, 95% CI = 0.226–0.858, *P* = 0.016), and the CC + TT genotype was 2.247 times more likely to be susceptible than the CT genotype (OR = 2.247, 95% CI = 1.192–4.234, *P* = 0.012). All positive results were visualized as Figure 1, and all results are shown in Supplementary Tables S2–S5.

### 3.3 Haplotype analysis of EGFR

Possible haplotypes at the rs2227983, rs2293347, and rs884225 SNPs of the EGFR gene were identified by SHEsis online software (Shi and He, 2023), including ACC, ACT, ATT, GCC, GCT, and GTT (Table 4). The GTC and ATC haplotypes were excluded from analysis due to low frequency, which precluded reliable conformation assessment. The ATT haplotype may act as a protective factor, reducing LC susceptibility risk (OR = 0.640, 95% CI = 0.465–0.881, *P* = 0.006); ATT and GTT haplotypes may be related to reduce the risk of LUAD susceptibility as protective factors (OR = 0.625 and 0.429, 95% CI = 0.431–0.906 and 0.208–0.887, *P* = 0.013 and 0.019, respectively); and GCC haplotype may be associated with a reduced risk of LUAD susceptibility as a risk factor (OR = 1.454, 95% CI = 1.029–2.056, *P* = 0.034); ATT haplotype may be associated with decreased risk of susceptibility to SCLC (OR = 0.596, 95% CI = 0.359–0.990, *P* = 0.044); ACT haplotype may be associated with associated with increased risk of SCLC susceptibility (OR = 1.820, 95% CI = 1.040–3.182, *P* = 0.034); all haplotypes were not associated with risk of LUSC susceptibility (all *P* > 0.05). However, after correction for multiple testing, only the ATT haplotype association in the HC vs. LC group remained significant (Bonferroni correction, *α* = 0.0083), and the associations of the other haplotypes may be false positives or need to be verified with larger samples.

**TABLE 4:** Haplotype analysis of EGFR gene in Chinese patients with Lung cancer.

Haplotype	ACC	ACT	ATT	GCC	GCT	GTT
HC vs. LC						
OR (95% CI)	1.116 (0.752–1.658)	1.255 (0.828–1.902)	0.640 (0.465–0.881)	1.195 (0.875–1.631)	1.336 (0.867–2.060)	0.669 (0.386–1.158)
*χ* ^2^	0.299	1.146	7.539	1.253	1.729	2.085
*P-*value	0.585	0.284	0.006*	0.263	0.188	0.149
HC vs. LUAD						
OR (95% CI)	1.099 (0.703–1.719)	1.073 (0.665–1.732)	0.625 (0.431–0.906)	1.454 (1.029–2.056)	1.339 (0.826–2.172)	0.429 (0.208–0.887)
*χ* ^2^	0.173	0.083	6.224	4.521	1.411	5.484
*P*-value	0.678	0.773	**0.013**	**0.034**	0.235	**0.019**
HC vs. LUSC						
OR (95% CI)	1.245 (0.730–2.125)	1.232 (0.697–2.179)	0.686 (0.434–1.086)	0.952 (0.609–1.487)	1.493 (0.843–2.646)	0.765 (0.350–1.670)
*χ* ^2^	0.649	0.515	2.597	0.047	1.904	0.456
*P-*value	0.420	0.473	0.107	0.828	0.168	0.500
HC vs. SCLC						
OR (95% CI)	0.760 (0.399–1.450)	1.820 (1.040–3.182)	0.596 (0.359–0.990)	0.974 (0.606–1.565)	1.063 (0.550–2.056)	1.115 (0.528–2.353)
*χ* ^2^	0.694	4.492	4.051	0.012	0.033	0.081
*P-*value	0.405	**0.034**	**0.044**	0.913	0.856	0.776

Data was presented by median (inter-quartile range, P25-P75). The *p*-values were calculated by Pearson’s chi-square test. *Multiple tests corrected for haplotype analysis *p*-values are still significant (using Bonferroni correction, *α* = 0.0083). Bold value indicates statistical significance.

### 3.4 Relationship between EGFR gene polymorphism and prognosis of LC

**FIGURE 2:**
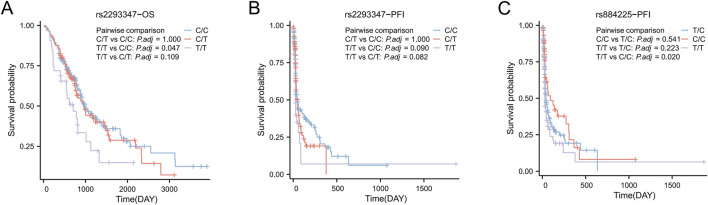
Kaplan–Meier survival curves of Progression Free Interval (PFI) and overall survival (OS) for all LC patients. **(A,B)** OS and PFI for LC patients with rs2293347 polymorphism **(C)** PFI for LC patients with rs884225 polymorphism.

The median overall survival (OS) of all LC patients was 938 days, and rs2293347-TT had a significantly shorter OS compared to CC (710 days vs. 1,014 days, *P* = 0.047) (Figure 2A). The median Progression Free Interval (PFI) for all LC patients was 41 days, and rs2293347-TT had a shortened PFI compared to CC (25 days vs. 47 days, *P* = 0.090) but the difference was not statistically significant (Figure 2B), rs884225-TT had a significantly shorter PFI compared to CC (28 days vs. 93 days, *P* = 0.022) (Figure 2C).

In univariate Cox analysis, rs2293347-TT was related to lower OS and PFI (HR = 1.810, 95% CI = 1.133–2.892, *P* = 0.013; HR = 1.744, 95% CI = 1.091–2.787, *P* = 0.020). Multivariate Cox analysis showed that rs2293347-TT was an independent adverse factor for OS (HR = 1.903, 95% CI = 1.176–3.078, *P* = 0.009), but not an independent adverse factor for PFI (HR = 1.229, 95% CI = 0.660–2.291, *P* = 0.516) (Tables 5, 6).

**TABLE 5:** Univariate and multivariate analyses of OS in LC patients.

Characteristics	OS			
	Univariate analysis		Multivariate analysis	
	HR (95% CI)	*P*-value	HR (95% CI)	*P*-value
Age	1.012 (0.994–1.031)	0.197		
Male/Female	0.562 (0.401–0.786)	<0.001	0.900 (0.565–1.434)	0.659
Smoking status: Yes/No	0.608 (0.447–0.826)	0.001	0.927 (0.565–1.522)	0.766
Drinking status: Yes/No	1.474 (1.073–2.026)	0.017	1.473 (0.956–2.267)	0.079
Stage:IV	—	—		
I	0.346 (0.110–1.093)	0.070	0.755 (0.229–2.492)	0.645
II	0.187 (0.046–0.761)	0.019	0.320 (0.077–1.334)	0.118
III	0.710 (0.492–1.023)	0.066	0.854 (0.581–1.255)	0.421
Treatment: chemotherapy	—	—		
Targeted therapy	1.079 (0.717–1.625)	0.716		
Biologically targeted therapy	0.800 (0.516–1.242)	0.321		
Histology: LUSC	—	—		
LUAD	1.183 (0.816–1.716)	0.375	1.233 (0.820–1.854)	0.315
SCLC	2.101 (1.444–3.055)	<0.001	1.835 (1.211–2.782)	**0.004**
Primary therapy outcome: PD	—	—		
PR	3.152 (2.087–4.760)	<0.001	2.964 (1.922–4.569)	**< 0.001**
SD	0.955 (0.569–1.604)	0.863	0.948 (0.557–1.612)	0.843
Cancerous site: Right	—	—		
Left	1.031 (0.749–1.420)	0.852		
Both	0.820 (0.202–3.335)	0.782		
Degree of tumor differentiation: Poorly	—	—		
Moderately	0.747 (0.428–1.301)	0.303		
High	0.864 (0.602–1.239)	0.426		
rs2227983: GG	—	—		
AA	0.880 (0.615–1.261)	0.486		
GA	0.799 (0.519–1.228)	0.306		
rs2293347: CC	—	—		
CT	1.098 (0.785–1.535)	0.587	1.203 (0.850–1.703)	0.297
TT	1.810 (1.133–2.892)	**0.013**	1.903 (1.176–3.078)	**0.009**
rs884225: TC	—	—		
CC	0.904 (0.600–1.363)	0.631		
TT	1.286 (0.911–1.816)	0.152		

CI, confidence interval; LUAD, lung adenocarcinoma; LUSC, lung squamous carcinoma; SCLC, small cell lung cancer; HR, hazard ratio; OS, overall survival; PR, partial response; SD, stable disease; PD, progressive disease. Bold value indicates statistical significance.

**TABLE 6:** Univariate and multivariate analyses of PFI in LC patients.

Characteristics	PFI			
	Univariate analysis		Multivariate analysis	
	HR (95% CI)	*P*-value	HR (95% CI)	*P*-value
Age	1.011 (0.993–1.030)	0.220		
Male/Female	0.496 (0.353–0.697)	<0.001	0.887 (0.534–1.474)	0.644
Smoking status: Yes/No	0.585 (0.431–0.795)	<0.001	0.914 (0.520–1.605)	0.754
Drinking status: Yes/No	0.700 (0.509–0.963)	0.029	0.841 (0.510–1.387)	0.497
Stage: IV	—	—		
I	0.372 (0.118–1.174)	0.092	1.490 (0.343–6.470)	0.594
II	0.240 (0.059–0.978)	0.046	0.381 (0.085–1.712)	0.208
III	0.847 (0.588–1.219)	0.371	1.079 (0.714–1.629)	0.718
Treatment: chemotherapy	—	—		
Targeted therapy	0.540 (0.316–0.922)	0.024	0.752 (0.391–1.449)	0.395
Biologically targeted therapy	0.953 (0.633–1.433)	0.816	1.027 (0.641–1.645)	0.913
Histology: LUSC	—	—		
LUAD	0.625 (0.428–0.914)	0.015	0.682 (0.424–1.096)	0.114
SCLC	1.448 (0.939–2.234)	0.094	1.229 (0.755–1.999)	0.407
Primary therapy outcome: PD	—	—		
PR	2.623 (1.741–3.950)	**< 0.001**	2.180 (1.392–3.412)	**< 0.001**
SD	1.072 (0.643–1.786)	0.790	1.107 (0.627–1.955)	0.727
Cancerous site: Right	—	—		
Left	1.073 (0.780–1.477)	0.666		
Both	0.716 (0.176–2.909)	0.640		
Degree of tumor differentiation: Poorly	—	—		
Moderately	0.945 (0.660–1.354)	0.758		
High	0.677 (0.388–1.181)	0.170		
rs2227983: GG	—	—		
AA	0.765 (0.521–1.123)	0.172		
GA	1.191 (0.831–1.708)	0.340		
rs2293347: CC	—	—		
CT	1.098 (0.783–1.540)	0.588	0.879 (0.589–1.311)	0.527
TT	1.744 (1.091–2.787)	**0.020**	1.229 (0.660–2.291)	0.516
rs884225: TC	—	—		
CC	0.762 (0.504–1.153)	0.199	0.810 (0.502–1.309)	0.390
TT	1.377 (0.978–1.939)	0.067	1.343 (0.868–2.078)	0.186

CI, confidence interval; LUAD, lung adenocarcinoma; LUSC, lung squamous carcinoma; SCLC, small cell lung cancer; HR, hazard ratio; PFI, progression free interval; PR, partial response; SD, stable disease; PD, progressive disease. Bold value indicates statistical significance.

### 3.5 Association between EGFR gene polymorphisms and LC tissue protein levels

**FIGURE 3:**
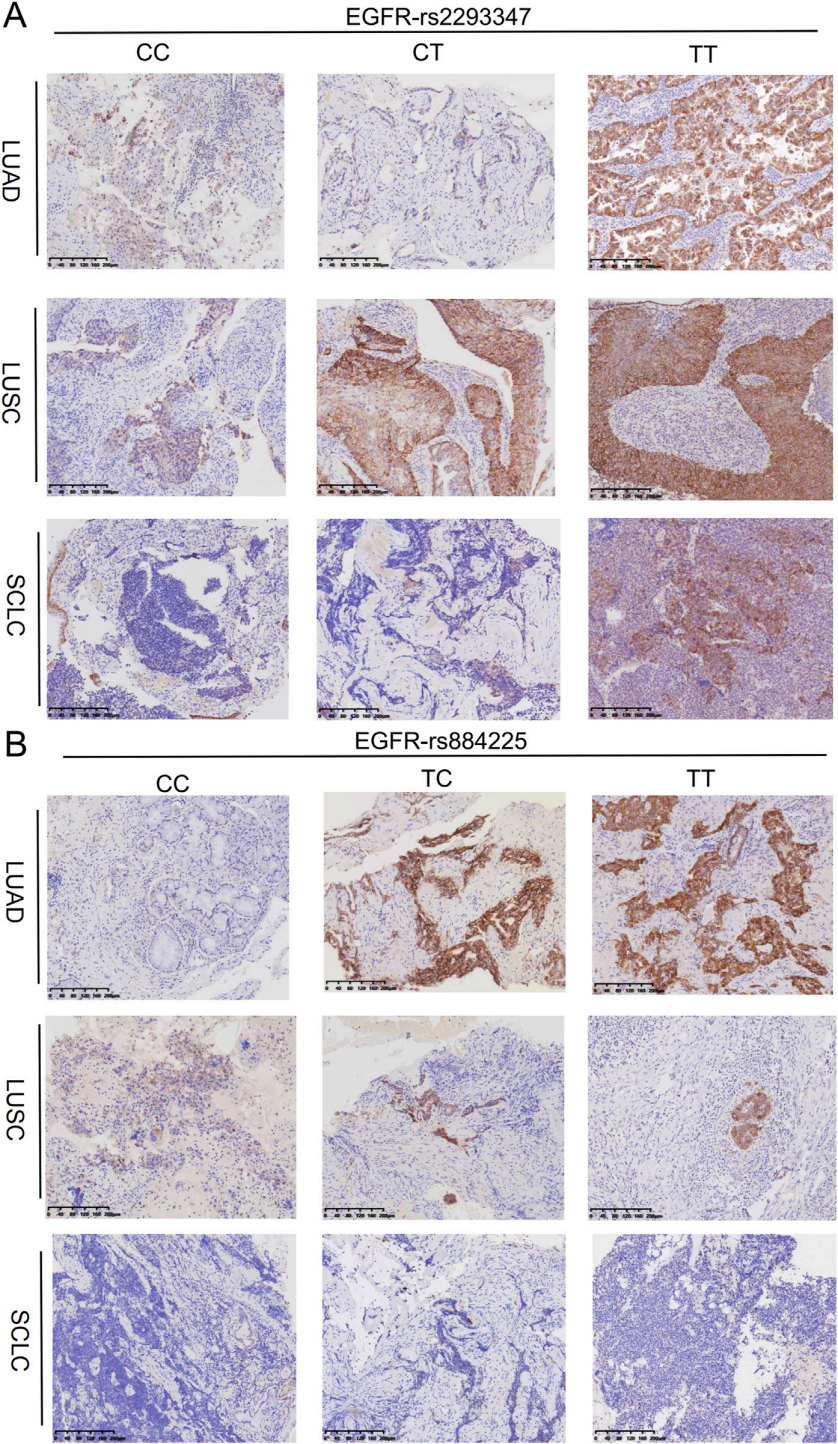
Relationship between protein expression and genotype of EGFR in LC patients. **(A)** rs2293347 **(B)** rs884225.

**FIGURE 4:**
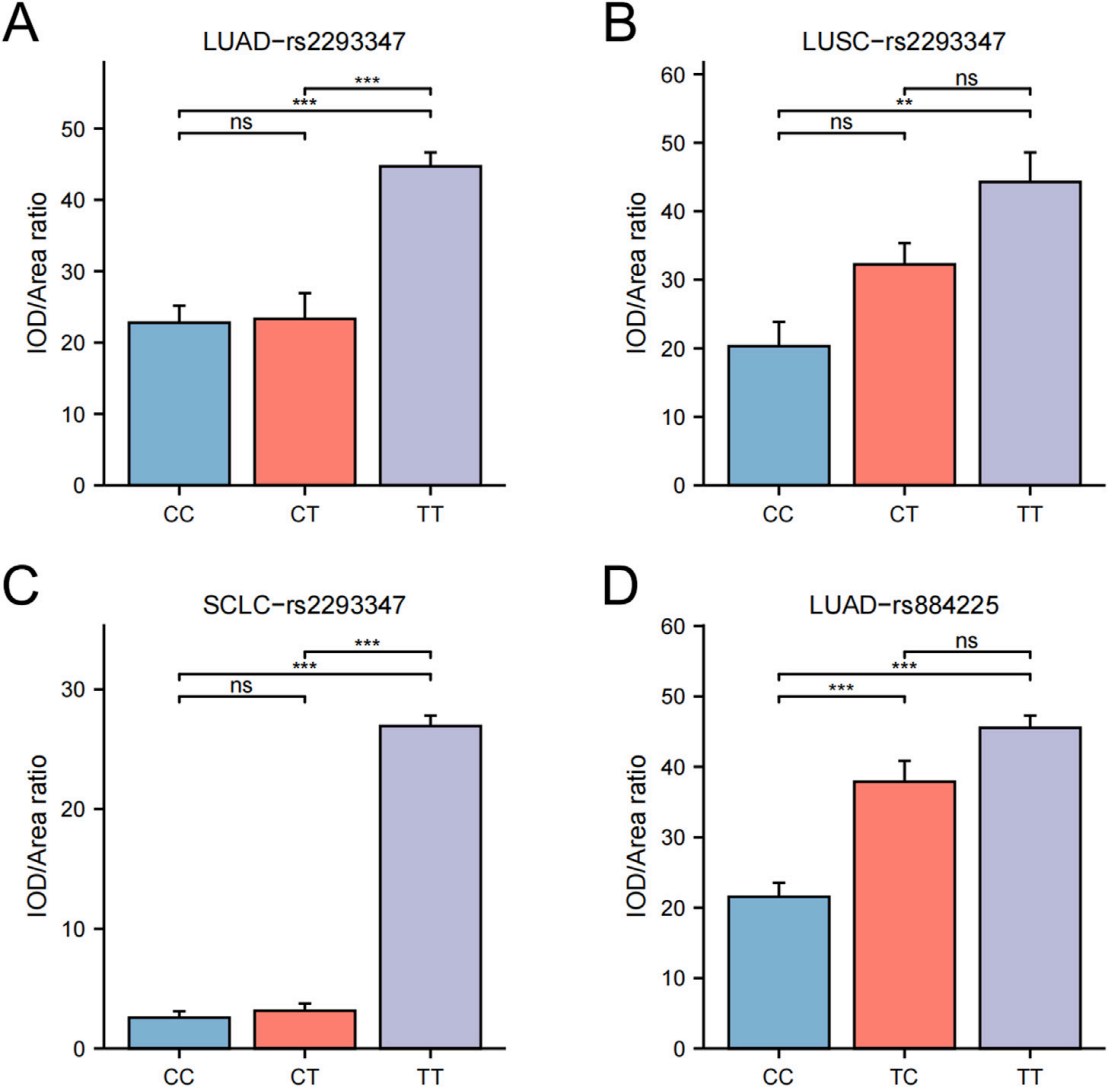
Differential statistics of EGFR genotypes and protein levels in LC patients. **(A)** rs2293347-LUAD **(B)** rs2293347-LUSC **(C)** rs2293347-SCLC **(D)** rs884225-LUAD.

Upon categorizing and examining the immunohistochemical outcomes by genotype, it was discovered that rs2293347-TT protein’s expression peaked across all LC histological types, with TT and CT surpassing CC (Figures 3A, 4A–C), and that rs884225-TT had the highest protein level in the LUAD population, with both TT and CT exceeding CC (Figures 3B, 4D). In addition, rs2227983 in all histological types and rs88425 in LUSC and SCLC histological types did not differ in genotype protein levels.

## 4 Discussion

To our knowledge, this is the first clinical research to check into the susceptibility to EGFR gene polymorphisms in Chinese patients with LC and to explore the relationship between EGFR protein levels in their lung cancer tissues. We found that EGFR gene polymorphisms were significantly associated with susceptibility and prognosis of LC and its histological types and were responsible for influencing the expression of protein levels.

With extensive research in molecular genetics, a variety of genetic and epigenetic changes have been potentially associated with the risk of developing LC (Zhu et al., 2015). The rs2293347 resides in exon 25, the C-terminal structural segment of the EGFR gene’s regulatory area (Mitsudomi and Yatabe, 2010). This genetic variation, A→G, leads to the substitution of aspartic acid in codon 994 with an identical amino acid in exon 25 s coding region, a synonymous SNP. Such synonymous SNPs do not result in coding sequence changes and may not be involved in affecting the inherent biological function of the protein (Kimchi-Sarfaty et al., 2007), but may lead to changes in protein number, structure, activity and function by affecting mRNA stability, selective splicing and translation kinetics (Sauna et al., 2007). In the present study, there was a statistically significant difference in the frequency of distribution of the rs2293347 SNP of the EGFR gene between the HC population and LC patients, which was significantly correlated with LC susceptibility, and this correlation persisted in subsequent subgroup analysis of pathology types. This is consistent with the results of a Jordanian population-based study (Bashir et al., 2019). Anne’s research (Winther-Larsen et al., 2019) revealed a notable extension in both progression-free survival (PFS) and overall survival (OS) among NSCLC patients with the CT genotype, in contrast to those with the 181946C>T (rs2293347) CC genotype, with numerous prior studies indicating a link between this particular SNP and LC susceptibility. And several previous studies (Zhang et al., 2013; Winther-Larsen et al., 2015; Ma et al., 2009) have reported that this polymorphism is significantly associated with efficacy response in NSCLC patients treated with TKI. Our KM curves and Cox regression results showed that compared to CC and CT, TT was an independent prognostic risk factor, with a significant reduction in both PFI and OS. Also in this study, carrying the CC genotype at this SNP has the potential to increase the risk of susceptibility to LUAD, LUSC and SCLC, carrying the CT or TT genotypes may be protective against the risk of susceptibility to LUAD, and carrying the CT genotype may be protective against the risk of susceptibility to LUSC and SCLC. Our research also revealed a link between rs2293347 and the expression of EGFR protein in LUAD, LUSC, and SCLC tumors, as identified through immunohistochemical staining. Additionally, lung tumor tissues from LUAD, LUSC, and SCLC patients with the TT genotype exhibited notably elevated EGFR levels compared to those with CC and CT genotypes, and that EGFR over expression has been demonstrated to be involved in numerous EGFR over expression is involved in the formation and progression of many malignant solid tumors, which effectively validates the TT genotype as an independent prognostic risk factor in this study. In conclusion, SNP at the rs2293347 of the EGFR gene may have the potential to predict LC susceptibility and prognostic risk, and its TT genotype is a protective factor against susceptibility to LC, but it may contribute to the increased worse prognosis risk of LC by inducing the upregulation of EGFR protein expression. Furthermore, considering the proximity of the rs2293347 SNP to the tyrosine kinase structural domain (Mitsudomi and Yatabe, 2010) and its correlation with the efficacy response of TKIs, this SNP may be a good candidate for future clinical trials targeting the EGFR gene to improve the clinical outcomes of LC patients.

Previous studies of the rs884225 SNP in exon 28 of the EGFR gene have shown that the rs884225 SNP is associated with the risk of developing Adverse reaction (ADR) in TKIs-treated patients with advanced NSCLC in the Chinese population (Winther-Larsen et al., 2019). In addition, Fan et al. (2019) showed that rs884225 was significantly related to EGFR expression levels and contributed to the risk of susceptible NSCLC, which is consistent with our results. However, a study from a Jordanian population reported that the rs884225 SNP was not significantly related to LC susceptibility (Bashir et al., 2019). Our study showed that the rs884225 SNP was significantly associated with LUAD susceptibility and EGFR protein expression levels in LUAD tumor tissues. We hypothesize that the rs884225 SNP could act as a possible genetic indicator for forecasting the likelihood of developing LUAD. Its T allele and TT genotype may serve as effective protective factors for susceptibility, but its resulting elevated EGFR protein levels may be responsible for a worse prognosis.

EGFR gene mutations are particularly important in guiding clinical therapeutic regimens, but their detection is often limited by factors such as difficulty in sourcing tumour tissues, technical complexity, and high cost. EGFR SNP testing offers practical advantages as a prognostic/diagnostic marker due to its cost-effectiveness and minimal sample requirements. Meanwhile, Ma’s study (Ma et al., 2011) showed that rs2293347 was associated with the efficacy of gefitinib in advanced NSCLC patients, and Zhang et al. (2013) found that the rs2293347 affects the OS of patients with LC in a population in southern China, which are further evidences of the potential of EGFR polymorphisms to guide targeted therapeutic strategies and personalized medicine. The inclusion of testing for EGFR gene polymorphisms in lung cancer screening programmes, especially for high-risk populations, can help in the early identification of individuals carrying unfavourable variants, and the adoption of targeted lifestyle modifications and regular surveillance to reduce the risk of morbidity. Individualised treatment plans are developed based on the patient’s EGFR gene variant status, including initial targeted drug selection, monitoring drug responsiveness, predicting recurrence risk, and adjusting treatment strategies to maximise therapeutic benefits. However, the above results are only speculative based on our results and the current study, and it is important to study how changes in EGFR expression in Chinese patients affect LC susceptibility and prognosis. Regardless, this study provides valuable insight into the role of EGFR in LC development and prognosis. Additionally, it proposes new strategies to assess risk and tailor interventions for patients with different histological types of LC in China.

Our study and analysis have certain limitations that need to be clarified. Firstly, our study focused on populations and patient tissues, and PDX mice and lung cancer cells should be investigated in future studies to uncover EGFR SNPs. Second, population segmentation needs to be strengthened, e.g., by differentiating between specific drugs and modalities under different treatment modalities, as well as independent analyses for small cell lung cancer. Meanwhile, the number of cases for certain indicators has decreased due to the lack of basic clinical data. Finally, as a cross-sectional, single-center study, we need to conduct multicenter longitudinal studies for further validation.

## 5 Conclusion

EGFR rs2293347 (pan-subtype) and rs884225 (LUAD-specific) polymorphisms increase LC risk through elevated protein expression, with rs2293347-TT conferring worse survival. These genotype-protein correlations highlight their dual role as susceptibility markers and prognostic predictors in precision oncology.

## Data Availability

The datasets presented in this study can be found in online repositories. The names of the repository/repositories and accession number(s) can be found in the article/Supplementary Material.

## Supplementary material

SUPPLEMENT FIGURE 1 Capillary electrophoresis peak map of EGFR gene polymorphism. **(A)** rs222798 **(B)** rs2293347 **(C)** rs884225.
