# Supplementary material for: EGFR polymorphisms drive lung cancer risk and survival disparities: a genotype-expression-outcome cohort study
Source: Front Genet. 2025 May 14;16:1591539. doi: 10.3389/fgene.2025.1591539 (PMC12116501; doi:10.3389/fgene.2025.1591539)
Supplement: Supplementary file 1 [file Supplementaryfile1.docx]

| Supplement Table 1: The specific PCR primer designed for the amplification ot the EGFR gene | | | |
| --- | --- | --- | --- |
| SNP ID | Primer probe sequences (5'-3') | Type | Length |
| rs2227983G/A | TGCTGGGGCCCGGAGCCAAG | identifying primer | 20bp |
|  | TGCTGGGGCCCGGAGCCGAA | identifying primer | 20bp |
|  | TGCTGGGGCCCGGAGCCGAT | identifying primer | 20bp |
|  | TGCTGGGGCCCGGAGCCGAC | identifying primer | 20bp |
|  | GGACTGCGTCTCTTGCCGGA | universal primer | 20bp |
| rs2293347C/T | ATCAGGGCACGGTAGAAGTTGTAG | identifying primer | 24bp |
|  | ATCAGGGCACGGTAGAAGTTGTAA | identifying primer | 24bp |
|  | TCTGTAGGACTTGGCAAATGCAT | universal primer | 23bo |
| rs884225T/C | CATGACAGCAAGACAGGGTCA | identifying primer | 21bp |
|  | CATGACAGCAAGACAGGGTCG | identifying primer | 21bp |
|  | CATGACAGCAAGACAGGGTCC | identifying primer | 21bp |
|  | GCATACTGAGTTTCAAAACAATGGAATG | universal primer | 28bp |

| Supplement Table 2: Logistic regression analysis of the genetic model (HC vs. LC) | | | | | |
| --- | --- | --- | --- | --- | --- |
| SNP IDs | genetic model | HC | LC | OR (95% CI) | P value |
| rs2227983 | GG | 34(22.7) | 71(23.7) | —— |  |
|  | GA | 68(45.3) | 154(51.3) | 1.085(0.659-1.785) | 0.750 |
|  | AA | 48(32.0) | 75(25.0) | 0.748(0.433-1.292) | 0.298 |
|  | G | 136(45.3) | 296(49.3) | —— |  |
|  | A | 164(54.7) | 304(50.7) | 0.852(0.645-1.125) | 0.258 |
|  | GG+GA vs. AA | 102(68.0) | 225(75.0) | 1.412(0.917-2.173) | 0.117 |
|  | AA+GA vs. GG | 116(77.3) | 229(76.3) | 0.945(0.593-1.506) | 0.813 |
|  | GG+AA vs. GA | 82(54.7) | 146(48.7) | 0.786(0.531-1.165) | 0.230 |
| rs2293347 | CC | 59(39.3) | 171(57.0) | —— |  |
|  | CT | 71(47.3) | 97(32.3) | 0.471(0.308-0.722) | <0.001 |
|  | TT | 20(13.3) | 32(10.7) | 0.552(0.293-1.039) | 0.065 |
|  | C | 189(63.0) | 439(73.2) | —— |  |
|  | T | 111(37.0) | 161(26.8) | 0.624(0.465-0.839) | **0.002** |
|  | CC+CT vs. TT | 130(86.7) | 268(89.3) | 1.288(0.709-2.340) | 0.405 |
|  | TT+CT vs. CC | 91(60.7) | 129(43.0) | 0.489(0.328-0.729) | <0.001 |
|  | CC+TT vs. CT | 79(52.7) | 203(67.7) | 1.881(1.259-2.810) | **0.002** |
| rs884225 | TT | 49(32.7) | 85(28.3) | —— |  |
|  | TC | 80(53.3) | 154(51.3) | 1.110(0.712-1.729) | 0.645 |
|  | CC | 21(14.0) | 61(20.3) | 1.675(0.912-3.075) | 0.096 |
|  | T | 178(59.3) | 324(54.0) | —— |  |
|  | C | 122(40.7) | 276(46.0) | 1.243(0.939-1.646) | 0.129 |
|  | TT+TC vs. CC | 129(86.0) | 239(79.7) | 0.638(0.372-1.095) | 0.103 |
|  | CC+TC vs. TT | 101(67.3) | 215(71.7) | 1.227(0.803-1.875) | 0.344 |
|  | TT+CC vs. TC | 70(46.7) | 146(48.7) | 1.083(0.732-1.604) | 0.689 |
|  |  |  |  |  |  |
| Supplement Table 3: Logistic regression analysis of the genetic model (HC vs. LUAD) | | | | | |
| SNP IDs | genetic model | HC | LUAD | OR (95% CI) | P value |
| rs2227983 | GG | 34(22.7) | 39(24.4) | —— | |
|  | GA | 68(45.3) | 87(54.4) | 1.115(0.638-1.950) | 0.702 |
|  | AA | 48(32.0) | 34(21.3) | 0.618(0.327-1.166) | 0.137 |
|  | G | 136(45.3) | 165(51.6) | —— | |
|  | A | 164(54.7) | 155(48.4) | 0.779(0.568-1.068) | 0.121 |
|  | GG+GA vs. AA | 102(68.0) | 126(78.8) | 1.744(1.046-2.907) | **0.033** |
|  | AA+GA vs. GG | 116(77.3) | 121(75.6) | 0.909(0.538-1.538) | 0.723 |
|  | GG+AA vs. GA | 82(54.7) | 73(45.6) | 0.696(0.445-1.088) | 0.112 |
| rs2293347 | CC | 59(39.3) | 94(58.8) | —— | |
|  | CT | 71(47.3) | 55(34.4) | 0.486(0.301-0.785) | **0.003** |
|  | TT | 20(13.3) | 11(6.9) | 0.345(0.154-0.772) | **0.010** |
|  | C | 189(63.0) | 243(75.9) | —— | |
|  | T | 111(37.0) | 77(24.1) | 0.540(0.381-0.764) | <0.001 |
|  | CC+CT vs. TT | 130(86.7) | 149(93.1) | 2.084(0.963-4.512) | 0.062 |
|  | TT+CT vs. CC | 91(60.7) | 66(41.3) | 0.455(0.289-0.717) | <0.001 |
|  | CC+TT vs. CT | 79(52.7) | 105(65.6) | 1.716(1.086-2.711) | **0.021** |
| rs884225 | TT | 49(32.7) | 40(25.0) | —— | |
|  | TC | 80(53.3) | 81(50.6) | 1.240(0.738-2.085) | 0.416 |
|  | CC | 21(14.0) | 39(24.4) | 2.275(1.158-4.469) | **0.017** |
|  | T | 178(59.3) | 161(50.3) | —— | |
|  | C | 122(40.7) | 159(49.7) | 1.441(1.048-1.980) | **0.024** |
|  | TT+TC vs. CC | 129(86.0) | 121(75.6) | 0.505(0.281-0.907) | **0.022** |
|  | CC+TC vs. TT | 101(67.3) | 120(75.0) | 1.455(0.888-2.386) | 0.137 |
|  | TT+CC vs. TC | 70(46.7) | 79(49.4) | 1.115(0.714-1.741) | 0.633 |
|  |  |  |  |  |  |
| Supplement Table 4: Logistic regression analysis of the genetic model (HC vs. LUSC) | | | | | |
| SNP IDs | genetic model | HC | LUSC | OR (95% CI) | P value |
| rs2227983 | GG | 34(22.7) | 19(24.7) | —— | |
|  | GA | 68(45.3) | 35(45.5) | 0.921(0.460-1.843) | 0.816 |
|  | AA | 48(32.0) | 23(29.9) | 0.857(0.405-1.815) | 0.688 |
|  | G | 136(45.3) | 73(47.4) | —— | |
|  | A | 164(54.7) | 81(52.6) | 0.920(0.623-1.358) | 0.675 |
|  | GG+GA vs. AA | 102(68.0) | 54(70.1) | 1.105(0.608-2.006) | 0.743 |
|  | AA+GA vs. GG | 116(77.3) | 58(75.3) | 0.895(0.470-1.703) | 0.735 |
|  | GG+AA vs. GA | 82(54.7) | 42(54.5) | 0.995(0.573-1.728) | 0.986 |
| rs2293347 | CC | 59(39.3) | 43(55.8) | —— | |
|  | CT | 71(47.3) | 24(31.2) | 0.464(0.253-0.851) | **0.013** |
|  | TT | 20(13.3) | 10(13.0) | 0.686(0.292-1.613) | 0.388 |
|  | C | 189(63.0) | 110(71.4) | —— | |
|  | T | 111(37.0) | 44(28.6) | 0.681(0.447-1.038) | 0.074 |
|  | CC+CT vs. TT | 130(86.7) | 67(87.0) | 1.031(0.457-2.327) | 0.942 |
|  | TT+CT vs. CC | 91(60.7) | 34(44.2) | 0.513(0.294-0.894) | **0.019** |
|  | CC+TT vs. CT | 79(52.7) | 53(68.8) | 1.985(1.112-3.541) | **0.020** |
| rs884225 | TT | 49(32.7) | 25(32.5) | —— | |
|  | TC | 80(53.3) | 38(49.4) | 0.931(0.502-1.726) | 0.820 |
|  | CC | 21(14.0) | 14(18.2) | 1.307(0.570-2.997) | 0.528 |
|  | T | 178(59.3) | 88(57.1) | —— | |
|  | C | 122(40.7) | 66(42.9) | 1.094(0.738-1.622) | 0.654 |
|  | TT+TC vs. CC | 129(86.0) | 63(81.8) | 0.733(0.349-1.536) | 0.410 |
|  | CC+TC vs. TT | 101(67.3) | 52(67.5) | 1.009(0.561-1.814) | 0.976 |
|  | TT+CC vs. TC | 70(46.7) | 39(50.6) | 1.173(0.677-2.033) | 0.570 |
|  |  |  |  |  |  |
| Supplement Table 4: Logistic regression analysis of the genetic model (HC vs. SCLC) | | | | | |
| SNP IDs | genetic model | HC | SCLC | OR (95% CI) | P value |
| rs2227983 | GG | 34(22.7) | 13(20.6) | —— | |
|  | GA | 68(45.3) | 32(50.8) | 1.231(0.573-2.645) | 0.595 |
|  | AA | 48(32.0) | 18(28.6) | 0.981(0.424-2.267) | 0.964 |
|  | G | 136(45.3) | 58(46.0) | —— | |
|  | A | 164(54.7) | 68(54.0) | 0.972(0.640-1.476) | 0.895 |
|  | GG+GA vs. AA | 102(68.0) | 45(71.4) | 1.176(0.617-2.243) | 0.622 |
|  | AA+GA vs. GG | 116(77.3) | 50(79.4) | 1.127(0.549-2.316) | 0.744 |
|  | GG+AA vs. GA | 82(54.7) | 31(49.2) | 0.803(0.446-1.448) | 0.466 |
| rs2293347 | CC | 59(39.3) | 34(54.0) | —— | |
|  | CT | 71(47.3) | 18(28.6) | 0.440(0.226-0.858) | **0.016** |
|  | TT | 20(13.3) | 11(17.5) | 0.954(0.409-2.229) | 0.914 |
|  | C | 189(63.0) | 86(68.3) | —— | |
|  | T | 111(37.0) | 40(31.7) | 0.792(0.509-1.233) | 0.301 |
|  | CC+CT vs. TT | 130(86.7) | 52(82.5) | 0.727(0.326-1.623) | 0.437 |
|  | TT+CT vs. CC | 91(60.7) | 29(46.0) | 0.553(0.305-1.002) | 0.051 |
|  | CC+TT vs. CT | 79(52.7) | 45(71.4) | 2.247(1.192-4.234) | **0.012** |
| rs884225 | TT | 49(32.7) | 20(31.7) | —— | |
|  | TC | 80(53.3) | 35(55.6) | 1.072(0.557-2.062) | 0.835 |
|  | CC | 21(14.0) | 8(12.7) | 0.933(0.355-2.453) | 0.889 |
|  | T | 178(59.3) | 75(59.5) | —— | |
|  | C | 122(40.7) | 51(40.5) | 0.992(0.649-1.516) | 0.971 |
|  | TT+TC vs. CC | 129(86.0) | 55(87.3) | 1.119(0.467-2.680) | 0.800 |
|  | CC+TC vs. TT | 101(67.3) | 43(68.3) | 1.043(0.555-1.960) | 0.896 |
|  | TT+CC vs. TC | 70(46.7) | 28(44.4) | 0.914(0.506-1.652) | 0.767 |
